# Neural networks reveal novel gene signatures in Parkinson disease from single-nuclei transcriptomes

**DOI:** 10.1038/s41531-025-01147-0

**Published:** 2025-10-21

**Authors:** Michael R. Fiorini, Jialun Li, Edward A. Fon, Sali M. K. Farhan, Rhalena A. Thomas

**Affiliations:** 1 Department of Human Genetics, McGill University, Montreal, QC Canada; 2 The Montreal Neurological Institute-Hospital, McGill University, Montreal, QC Canada; 3 Department of Neurology and Neurosurgery, McGill University, Montreal, QC Canada

**Keywords:** Parkinson's disease, Computational biology and bioinformatics, Genetics

## Abstract

Parkinson disease (PD) is a progressive neurodegenerative disease with an incompletely understood genetic architecture that necessitates novel discovery methods. We introduce an explainable machine learning framework that uses single-cell/nuclei RNA sequencing (sc/snRNAseq) to identify molecular markers of diseased cells and nominate candidate genes for targeted genomic analysis. Application to four snRNAseq datasets characterizing the post-mortem midbrain identified cell type-specific gene sets that consistently distinguished PD from healthy cells across all datasets (mean balanced accuracy = 0.92) and highlighted ten novel candidate genes in PD. Among these, *GPC6* was identified as a marker of PD dopaminergic neurons and a member of the heparan sulfate proteoglycan family, implicated in the intracellular accumulation of α-synuclein preformed fibrils—a hallmark of PD. We further validated the enrichment of rare *GPC6* variants in PD across three case-control cohorts. This open-source framework is broadly applicable across diseases and promises to accelerate gene discovery in complex diseases.

## Introduction

Parkinson disease (PD) is a progressive neurodegenerative disease marked by motor symptoms such as bradykinesia, tremor, and balance issues^1^. Its pathological hallmarks include Lewy bodies^2^ and the loss of dopaminergic neurons (DaNeurons) in the substantia nigra pars compacta (SNpc)^3^, yet growing evidence suggests that diverse cell types, including microglia^4^, astrocytes^5^, and oligodendrocytes^6^, contribute to its pathogenesis. PD displays heritability estimates around 30%, with only 5–10% of cases attributed to monogenic variants in established genes^7,8^. The remaining cases likely result from the complex interplay between genetic risk and environmental exposures. Currently, known genes account for only 16–36% of PD heritability^9^, necessitating the use of novel methodologies to uncover elusive genetic determinants. Given that no disease-modifying treatments currently exist, understanding PD genetics is essential to advance our understanding of disease biology and facilitate the clinical translation of findings to targeted therapeutics.

Transcriptomics offers a way to bridge genetics and disease biology by revealing how differential gene expression (DGE) drives pathogenesis. Single-cell RNA sequencing (scRNAseq) is particularly insightful as it reveals DGE in individual cells within disease-relevant tissues. To date, this technology has revealed cell types vulnerable to degeneration in PD and has clarified their roles in disease progression^10–12^. Additionally, differentially expressed genes (DEG) identified by scRNAseq can nominate candidate genes for targeted genomic analyses to reveal novel associations that may have been overlooked in broader exome- or genome-wide analyses. However, the robust identification of DEGs in scRNAseq remains challenging. High costs limit the feasibility of profiling entire patient cohorts, which is essential to capture PD heterogeneity and ensure robust signals^13^. Furthermore, DEG detection is complicated by conflicting benchmarks on optimal statistical methods^14–16^.

The limitations associated with standard DGE methods, coupled with advancements in machine learning (ML) raises the question whether these state-of-the-art algorithms can identify robust changes in gene expression at a single-cell resolution. Specifically, ML classifiers can be trained to distinguish between the transcriptomes of diseased and healthy cells, a technique that has been previously applied to whole blood bulk RNA sequencing from individuals with PD and healthy controls^17^. Importantly, accurate disease classifiers open avenues for deeper investigation into the underlying “reasons” for a particular cell being classified as diseased. While many ML classifiers operate as “black boxes”^18^, interpretable explainers like Local Interpretable Model-agnostic Explanations (LIME) reveal the most influential features driving a classification decision^19^. These LIME-identified features could provide valuable insights into the molecular characteristics of PD cells and nominate genes for targeted genomic analyses aimed at uncovering novel genetic associations.

In this work, we introduce a ML framework for identifying gene expression changes between diseased and healthy cells. We first evaluate four different ML classifiers for their ability to distinguish transcriptomes according to disease status. We then apply our approach to a pan-dataset analysis of single-nucleus RNA sequencing (snRNAseq) data from post-mortem midbrains of individuals with PD and controls, identifying transferrable molecular markers that distinguish PD cells.

## Results

### Neural network classifiers best predict disease status from single-nuclei transcriptomes

**Fig. 1:**
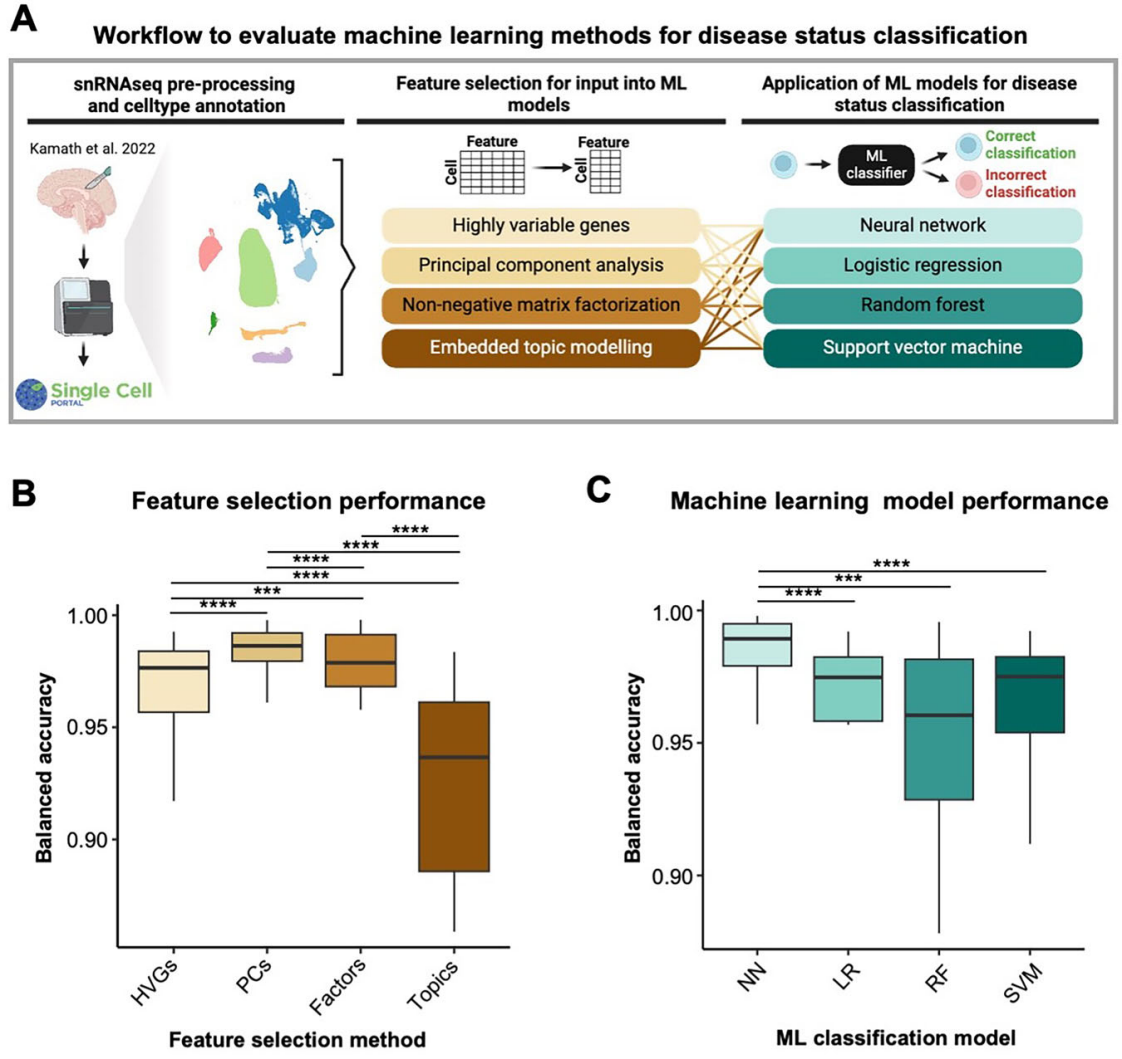
Evaluating different methods for classifying single-nuclei transcriptomes by disease status. **A** Schematic of the analysis workflow. For this analysis, we used publicly available single-nuclei RNA sequencing (snRNAseq) data from the post-mortem substantia nigra of individuals with Parkinson disease and controls prepared by Kamath et al. Four feature selection methods were independently applied to the snRNAseq data before being input into four distinct machine learning (ML) models for disease classification of individual cells. A total of 16 unique combinations of feature selection methods and ML models were tested, with each combination applied to classify seven distinct cell types independently and all cell types together. For each feature selection-ML model combination, we trained five independent models per cell type using five-fold cross validation (*n* = 128 models). **B** Boxplot showing the disease classification accuracy obtained using different feature selection methods. **C** Boxplot showing the disease classification accuracy obtained using different ML models. A Wilcoxon rank-sum test was used to compare mean balanced accuracies; non-significant comparisons are not shown. NN neural network, HVG highly variable gene, LR logistic regression, PC principal component, RF random forest, SVM support vector machine. *** *P* < 0.001; **** *P* < 0.0001.

To optimize the ML framework for classifying single-nuclei transcriptomes by disease status, we used a snRNAseq dataset from Kamath et al., profiling seven broad cell types in the substantia nigra of individuals with PD (*n* = 6) and controls (*n* = 8) (Fig. 1A). We evaluated 16 model combinations by pairing four feature selection methods—highly variable genes (HVG), principal component analysis (PCA), non-negative matrix factorization (NMF), and embedded topic modeling (ETM)—with four ML models—neural network (NN), logistic regression (LR), random forest (RF), and support vector machine (SVM)—across each cell type. On average, PCA yielded the highest overall classification accuracy (mean balanced accuracy = 0.984), followed by NMF (0.975), HVGs (0.965), and ETM (0.921) (Fig. 1B and Supplementary Fig. 1). Among the classifiers, NNs performed best (mean balanced accuracy = 0.984), followed by RF (0.955), LR (0.954), and SVM (0.952) (Fig. 1C). Although differences in performance across ML models and feature selection methods were modest, selecting higher-performing ML models offers distinct advantages for the purpose of decoding the models to identify key gene expression changes in PD. First, models with higher accuracy on unseen test data are, by definition, more likely to have captured gene expression patterns that better generalize to unseen cells, making their decoded features more robust. Second, our framework was designed to apply LIME only to correctly classified cells. Therefore, higher model accuracy increases the pool of cells available for interpretation, improving the reliability and generalizability of the resulting gene signatures.

Based on these results, we selected HVGs—genes that show variation across all cells, independent of disease status—for feature selection and NNs for disease classification in subsequent analyses. Although slightly less accurate than PCA and NMF, we selected the HVG feature selection method because it provides directly interpretable results upon decoding the classifiers to elucidate the most important features distinguishing PD cells. In contrast, decoding a model trained on PCA, for example, would return a ranked list of principal components, each of which represents a linear combination of many genes, making it difficult to assign biological meaning to specific gene contributions. Thus, selecting an appropriate machine learning framework for our application requires a careful balance between maximizing predictive performance and ensuring the biological interpretability of the input features driving classification decisions.

### Neural networks accurately identify Parkinson disease transcriptomes across midbrain cell types

To further assess the utility of machine learning classifiers in identifying gene expression signatures of distinct cell types in the PD midbrain, we began by accessing three publicly available snRNAseq datasets profiling post-mortem midbrain tissue from individuals with PD and controls: (i) Kamath et al., (ii) Wang et al., and (iii) Smajic et al. (Table 1). Specifically, the Kamath et al. and Wang et al. datasets were used for exploratory analyses to train cell type- and dataset-specific classifiers and identify gene signatures that distinguish PD from control cells, while the Smajic et al. dataset served as an independent validation set to evaluate the transferability of these gene signatures.

**Table 1:** Summary of single-nuclei RNA sequencing datasets characterizing Parkinson disease and healthy control midbrains

	*N* subjects		*N* cells					
Author	PD	Control	PD	Control	Genes per cell (median)	UMI per cell (median)	% mitochondrial genes (mean)	% ribosomal proteins (mean)
Kamath et al. ^10^	6	8	127,647	172,866	3259	7442	2.33	0.89
Wang et al. ^11^	5	6	21,890	26,367	1211	2065	5.40	0.61
Smajic et al. ^12^	6	5	17,995	22,289	2453	5279	1.49	0.91

*PD* Parkinson disease, *UMI* unique molecular identifier.

**Fig. 2:**
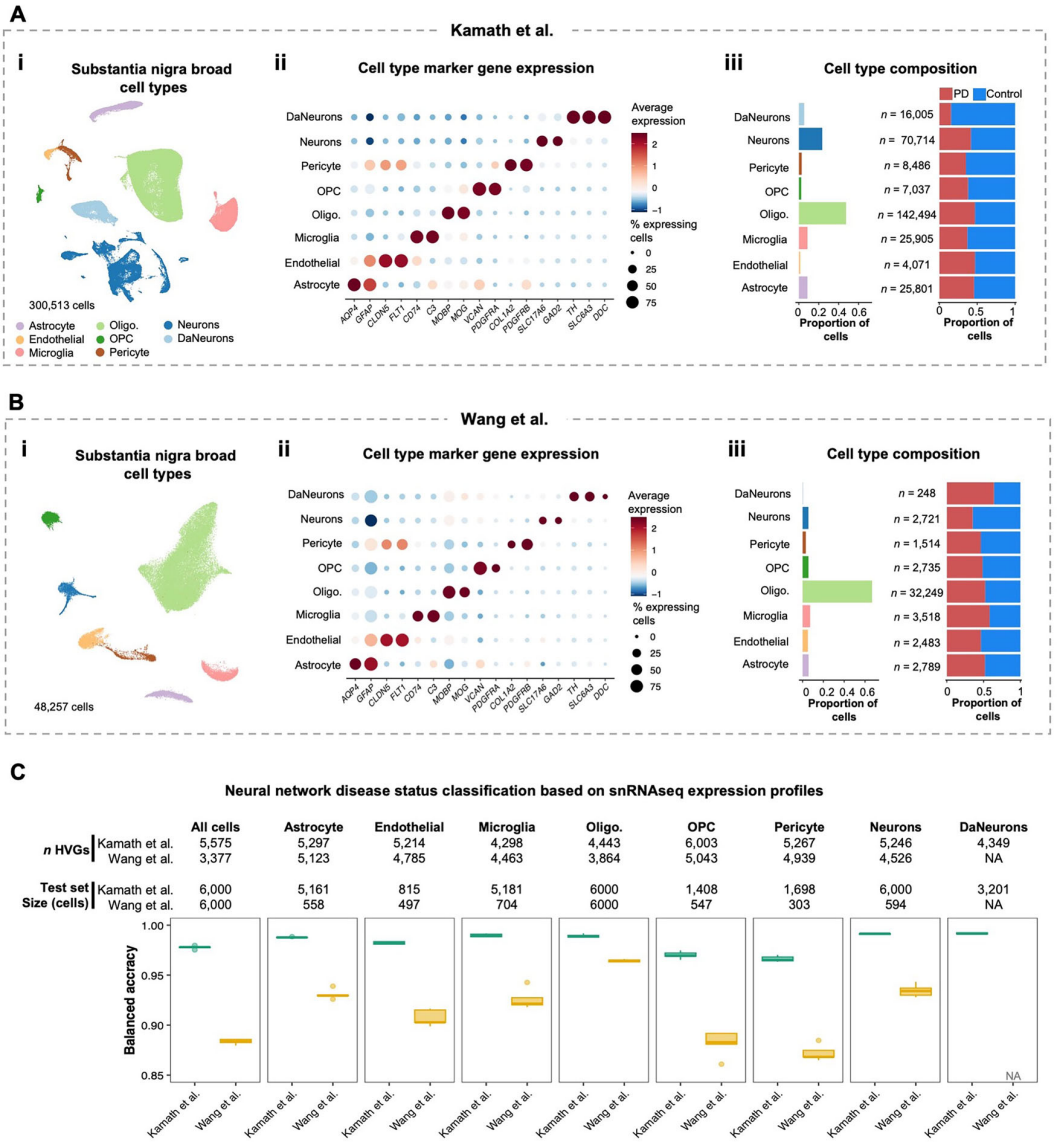
Application of neural networks to classify single-nuclei transcriptomes from the midbrain by disease status. **A**, **B** Characterization of the single-nuclei RNA sequencing (snRNAseq) datasets prepared by Kamath et al. and Wang et al., respectively. (i) Uniform manifold approximation and projection (UMAP) showing the annotated cell types comprising the midbrain. (ii) Dot plot showing the expression of literature-curated cell type marker genes. (iii) Bar plots showing the proportion of cells annotated to each cell type (left) and the distribution of cells from individuals with Parkinson disease (PD) and controls (right). The total number of cells per cell type is shown. **C** Boxplots showing the disease classification balanced accuracy of the neural network (NN) models. Independent NN classifiers were trained and evaluated for unique cell types from each dataset. Five-fold cross-validation on the training set resulted in five distinct models that were each independently applied to the test set for model evaluation. The number of cells in the test set reserved for NN evaluation is indicated at the top of the panels as well as the number of highly variable genes (HVG) input to the models. Dopaminergic neurons (DaNeurons) from Wang et al. were excluded due to insufficient cells for the ML test-train split workflow. oligo oligodendrocytes, OPC oligodendrocyte precursor cells.

We annotated each dataset to eight broad cell types—astrocytes, endothelial cells, microglia, oligodendrocytes, oligodendrocyte precursor cells (OPC), pericytes, neurons, and DaNeurons—based on published cell type annotations and the expression of known cell type marker genes (Fig. 2A, B and Supplementary Fig. 2B). Evaluating the transcriptional similarity of cell types across datasets revealed high inter cell-type replicability (mean area under the curve = 0.987) (Supplementary Fig. 3), supporting our pan-dataset approach. Importantly, too few DaNeurons were identified in the Wang et al. and Smajic et al. datasets (*n* = 248 and 57, respectively) to train our ML classifiers. This underrepresentation of DaNeurons may be attributed to their sparsity in the substantia nigra and the vulnerability of neurons to loss during enzymatic or mechanical dissociation in preparation of the single-cell suspension for sequencing^20,21^. Conversely, Kamath et al. enriched their dataset for neurons using fluorescence-activated cell sorting prior to sequencing, affording 16,005 DaNeurons; thus, initial analyses of DaNeurons were restricted to the Kamath et al. dataset.

Next, we identified HVGs within each of the seven broad cell types, as well as across all cell types combined, from the exploratory datasets (Kamath et al. and Wang et al.). The average number of cell-type specific HVGs was 5,015 and 4,678 for Kamath et al. and Wang et al., respectively (Fig. 2C). The HVG expression matrices were then used to train cell type- and dataset-specific NN classifiers for disease prediction. The average balanced accuracy on the held-out test sets for NNs trained on the Kamath et al. and Wang et al. datasets was 0.983 and 0.913, respectively (Fig. 2C). Further analyses revealed that the higher classification accuracy in the Kamath et al. dataset likely reflects its greater cell count and sequencing depth compared to Wang et al. (Supplementary FigS. 4–6). To verify that NN classifiers captured true PD-related expression patterns rather than noise, we progressively randomized disease labels and retrained the models. Across the cell type- and dataset-specific NNs, the mean balanced accuracy dropped from 0.948 with 0% randomization to 0.496 with 100% randomization, the latter being equivalent to chance performance (Supplementary Fig. 7). Taken together, these results demonstrate that NNs can effectively detect PD-associated changes in gene expression, enabling accurate disease status classification across all cell types in two distinct datasets and supporting our approach to leverage these models to identify molecular markers of PD in subsequent analyses.

### LIME identifies transcriptional markers of Parkinson disease cells consistent across datasets

**Fig. 3:**
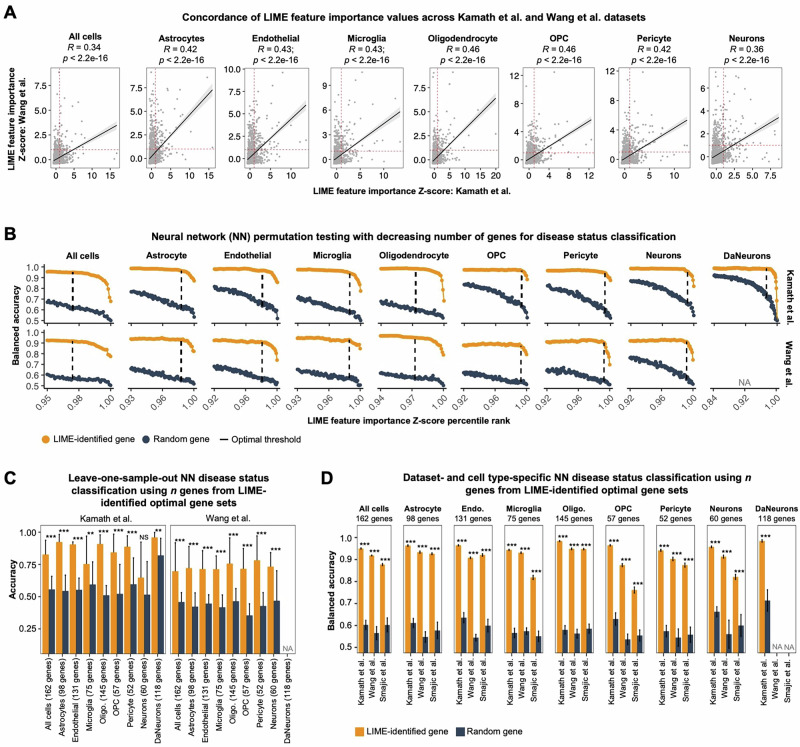
Application of LIME to identify genes influencing the classification decision by neural networks. **A** Scatter plots showing the cell type-specific correlations between the LIME feature importance Z-scores in the exploratory datasets. Pearson’s correlation coefficient (*R*) and the corresponding P-value are shown at the top of the panels. **B** Dot plots showing the neural network (NN) disease classification balanced accuracy obtained from permutation tests with decreasing feature counts used as input. HVGs with a mean LIME feature importance Z-score > 1.00 were incrementally eliminated based on their Z-score percentile rank until only the most important features remained. The same permutation tests were performed with an equal number of randomly selected genes as a benchmark. The dashed LIME indicates the optimal threshold: the number of input genes that maximized the discrepancy in balanced accuracy between using LIME-identified genes and random genes, across both Kamath et al. (top) and Wang et al. (bottom). **C** Bar plots showing the median NN accuracy using a leave-one-subject-out approach with LIME-identified genes or an equal number of randomly selected genes. Error bars represent the mean absolute deviation of the median accuracy across ten permutations for each subject. **D** Bar plots showing the dataset- and cell type specific-NN balanced accuracy when using the LIME-identified genes or an equal number of randomly selected genes. Error bars represent the standard deviation of the balanced accuracy across ten permutations. Wilcoxon rank-sum tests were used to compare model performance when using the LIME-identified genes versus randomly selected genes; a distinct set of random genes were used for each permutation. *P < 0.05; **P < 0.01; *** *P* < 0.001. DaNeurons dopaminergic neurons, NS not significant, oligo oligodendrocytes, OPC oligodendrocyte precursor cells.

To decode the “black box” of the NN classifiers, we applied LIME to reveal the most influential genes driving the classification of PD cells. In brief, LIME approximates the local decision boundary of a complex model with an interpretable linear model, assigning importance scores to individual features—in this case, genes—that most strongly influence each prediction. To evaluate the generalizability of LIME-identified features, we first calculated Pearson’s correlation between feature importance Z-scores from the two exploratory datasets. We observed medium strength correlations within each cell type, indicating consistency in a subset of key genes across datasets (Fig. 3A). Next, we compared NN-LIME to four well-established DGE methods, including the Wilcoxon rank-sum test, MAST, MAST under a mixed-effects framework, and pseudobulk-DESeq2 by identifying cell type-specific DEGs in each exploratory dataset and then calculating the proportion of overlapping genes between datasets (pan-dataset genes). NN-LIME (Z-score > 1.00) identified a significantly higher proportion of pan-dataset genes (10.25%) than the Wilcoxon rank-sum test (4.44%), MAST (3.88%), MAST under a mixed-effects framework (1.24%), and DESeq2 (0.92%) (Supplementary Fig. 8). These results suggest that our NN-LIME framework is particularly effective at identifying molecular markers of disease that generalize across datasets.

Next, we sought to identify genes consistently important for disease classification in both exploratory datasets. We first averaged cell type-specific LIME feature importance Z-scores across exploratory datasets for each HVG input to the NNs and selected genes with a mean Z-score > 1.00, yielding cell type-specific lists of key PD markers (Supplementary Fig. 9). To identify the minimum number of LIME-identified genes needed for accurate disease classification, we performed permutation testing in which we evaluated cell type- and dataset-specific NN classifiers using progressively fewer genes as input, removing features stepwise and assessing performance at each step until only the most influential genes remained (ranked by mean LIME Z-score). Additionally, we repeated this process using randomly selected genes to compare classification performance. Across permutations, NNs using LIME-identified genes consistently outperformed those using random genes (Fig. 3B). To determine the optimal gene sets for disease classification in each cell type, we identified the number of genes that maximized the performance gap between the NN classifiers using LIME-identified genes and random genes for both datasets (dashed line in Fig. 3B). The optimal gene sets for disease classification are listed in Supplementary Table 1: all cells = 162 genes; astrocytes = 98 genes; endothelial = 131 genes; microglia = 75 genes; oligodendrocytes = 145 genes; OPCs = 57 genes; neurons = 60 genes; DaNeurons = 118 genes.

To assess subject-level generalizability of the LIME optimal gene sets, we performed leave-one-subject-out NN classification using LIME-identified genes as input, training on all but one subject and testing on the held-out subject. The process was also repeated using randomly selected genes as input. Across both datasets, median NN classification accuracy was higher with LIME genes as input (0.833) than with random genes (0.632) (Fig. 3C). Furthermore, all cell type- and dataset-specific NNs achieved significantly higher classification accuracy than models using random genes, except for non-DaNeurons in the Kamath et al. dataset. Notably, for DaNeurons, NNs supplied with random genes achieved high accuracy (0.819), yet the models taking LIME genes as input still performed significantly better (0.958, Wilcoxon *P* = 3.00e-3), reinforcing that LIME effectively pinpointed the most relevant genes distinguishing PD cells across patients.

Finally, to test whether the cell type-specific LIME optimal gene sets were transferable to a third dataset (Smajic et al.), we re-trained NN classifiers for all three datasets using these genes as input and compared the performance to models using equally sized sets of randomly selected genes. In Kamath et al., Wang et al., and the Smajic et al. validation dataset, the average NN balanced accuracies across cell types using LIME genes as input were 0.962, 0.916, and 0.868, respectively, compared to 0.620, 0.554, and 0.578 with random genes (Fig. 3D). Across all datasets, classifiers using LIME genes significantly outperformed those using random genes (Supplementary Table 2). Together, these analyses demonstrate that the LIME optimal gene sets can effectively classify PD cells across three separate datasets, highlighting the robustness of the signals and the ability of our NN-LIME framework to identify generalizable molecular markers.

### LIME-identified genes are biologically relevant to Parkinson disease pathology

To assess the biological relevance of the LIME optimal gene sets we first performed gene set overrepresentation analyses. Across non-neuronal cell types, we observed an enrichment of processes related to the heat shock and unfolded protein response, consistent with their well-established role in PD (Supplementary Figs. 10–18)^13^. Microglia, oligodendrocytes, and OPCs showed enrichment for antigen presentation, supporting immune involvement in PD^22^, while astrocytes uniquely showed copper ion dysregulation, linked to dopamine metabolism and α-synuclein aggregation^23^. The LIME optimal gene set for neurons was enriched for genes involved in aggrephagy, while the DaNeuron gene set was enriched for terms suggesting synaptic dysfunction, which has been linked to the loss of this vulnerable cell type in PD^24^.

**Fig. 4:**
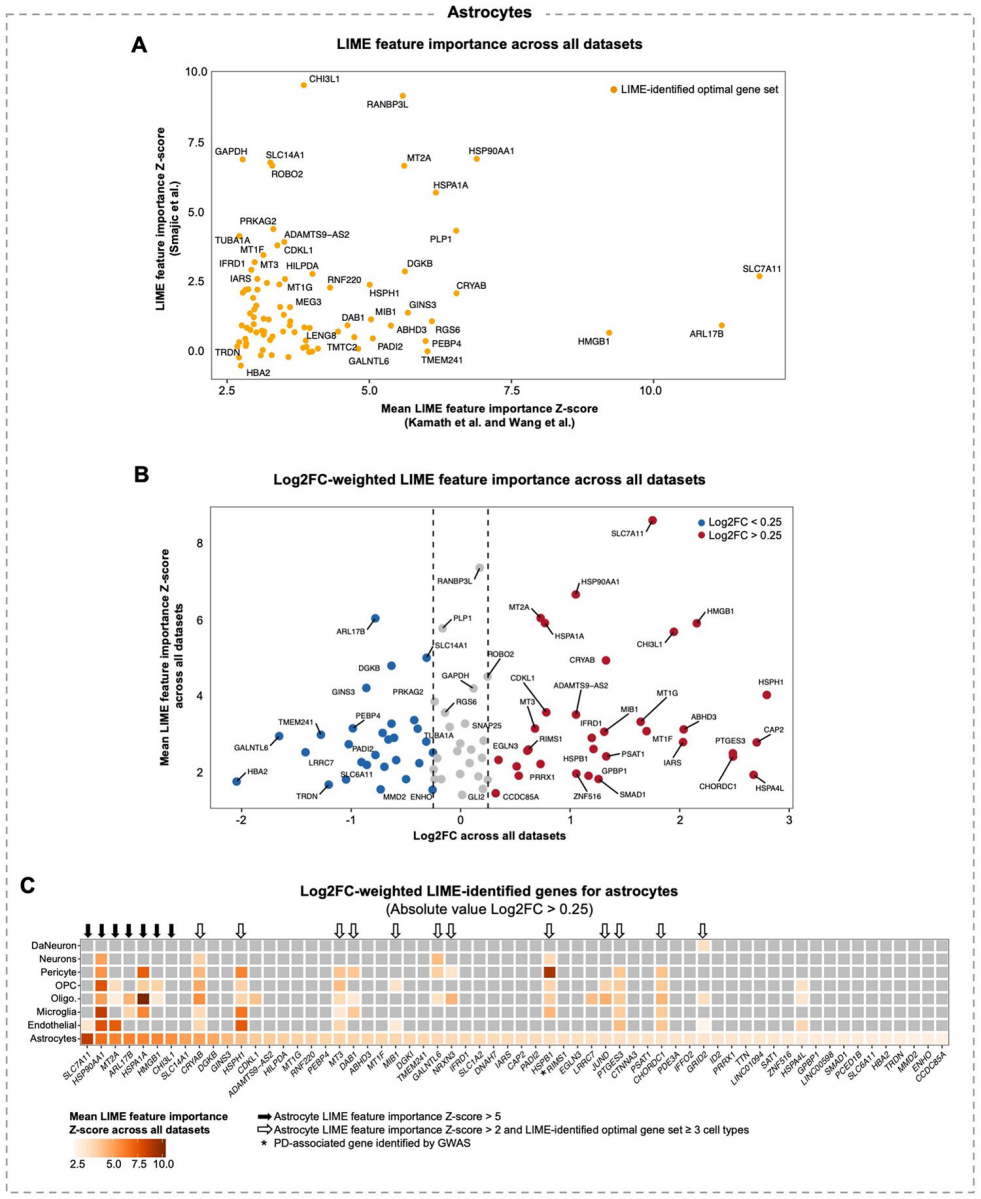
Prioritization of LIME-identified genes for characterizing Parkinson disease cells. This figure uses astrocytes as a case study to demonstrate our approach for identifying the most biologically relevant genes identified by LIME. **A** Scatter plot showing the LIME feature importance Z-score for genes in the LIME optimal gene set for astrocytes. **B** Volcano plot showing the mean LIME feature importance Z-score across the Kamath et al., Wang et al., and Smajic et al. datasets and the gene expression log2 fold-change (Log2FC) between Parkinson disease (PD) and control astrocytes across all datasets. Only genes from the LIME-identified optimal gene set for astrocytes are included. **C** Heatmap showing the mean LIME feature importance Z-scores for genes in the astrocyte optimal gene set with an absolute Log2FC > 0.25. Grey tiles indicate genes that were not included in the cell type-specific optimal gene set. The genes retained for subsequent analysis are labelled. oligo oligodendrocytes, OPC oligodendrocyte precursor cells.

We next employed a three-step prioritization strategy to nominate novel candidate genes for further investigation from the LIME optimal gene sets. To also explore known PD-associated genes, we retained LIME-identified genes previously implicated in PD via genome-wide association study (GWAS). We excluded mitochondrial DNA, ribosomal subunits, sex-linked genes, and non-coding RNAs from our gene lists. Figure 4 illustrates this prioritization process using astrocytes.

We first compared the LIME feature importance values from the Kamath et al. and Wang et al. datasets with those from the Smajic et al. validation dataset, identifying genes consistently important for distinguishing PD cells. In astrocytes, genes with consistently high LIME feature importance values included *HSP90AA1*, *HSPA1A*, *MT2A*, and *CRYAB*, all of which have been implicated in PD (Fig. 4A)^25–29^. Next, we integrated all three datasets and calculated cell type-specific Log2FC in gene expression between PD and control cells, retaining LIME genes with absolute Log2FC > 0.25. This reduced the astrocyte LIME-identified gene set from 98 to 62 genes (Fig. 4B). From these, we selected genes with a LIME feature importance Z-score > 5.00 in a particular cell type or genes with a Z-score > 2.00 in a particular cell type but identified by LIME in ≥ 3 cell types. This yielded seven highly informative genes for PD astrocytes—*SLC7A11*, *HSP90AA1*, *MT2A*, *ARL17B*, *HSPA1A*, *HMGB1*, and *CHI3L1*—and 12 genes with broader importance for distinguishing diseased cells across multiple cell types— *CRYAB*, *HSPH1*, *MT3*, *DAB1*, *MIB1*, *GALNTL6*, *NRXN3*, *HSPB1*, *JUND*, *PTGES3*, *CHORDC1*, and *GRID2* (Fig. 4C). Additionally, LIME identified *RIMS1*, which has been previously implicated in PD by GWAS^30,31^, as a key astrocyte marker.

We applied the same prioritization approach to the LIME optimal gene sets for all cell types (Supplementary Figs. 19–26; Supplementary Table 3). For DaNeurons, LIME scores and Log2FC were calculated using only the Kamath et al. dataset. This process yielded 66 unique genes across all cell types: 41 genes with high importance in a single cell type and 25 genes with high importance in ≥ 3 cell types (Supplementary Tables 4–5). These genes were the focus of subsequent analyses.

### Genetic analysis of LIME-identified features implicates multiple genes in Parkinson disease

**Fig. 5:**
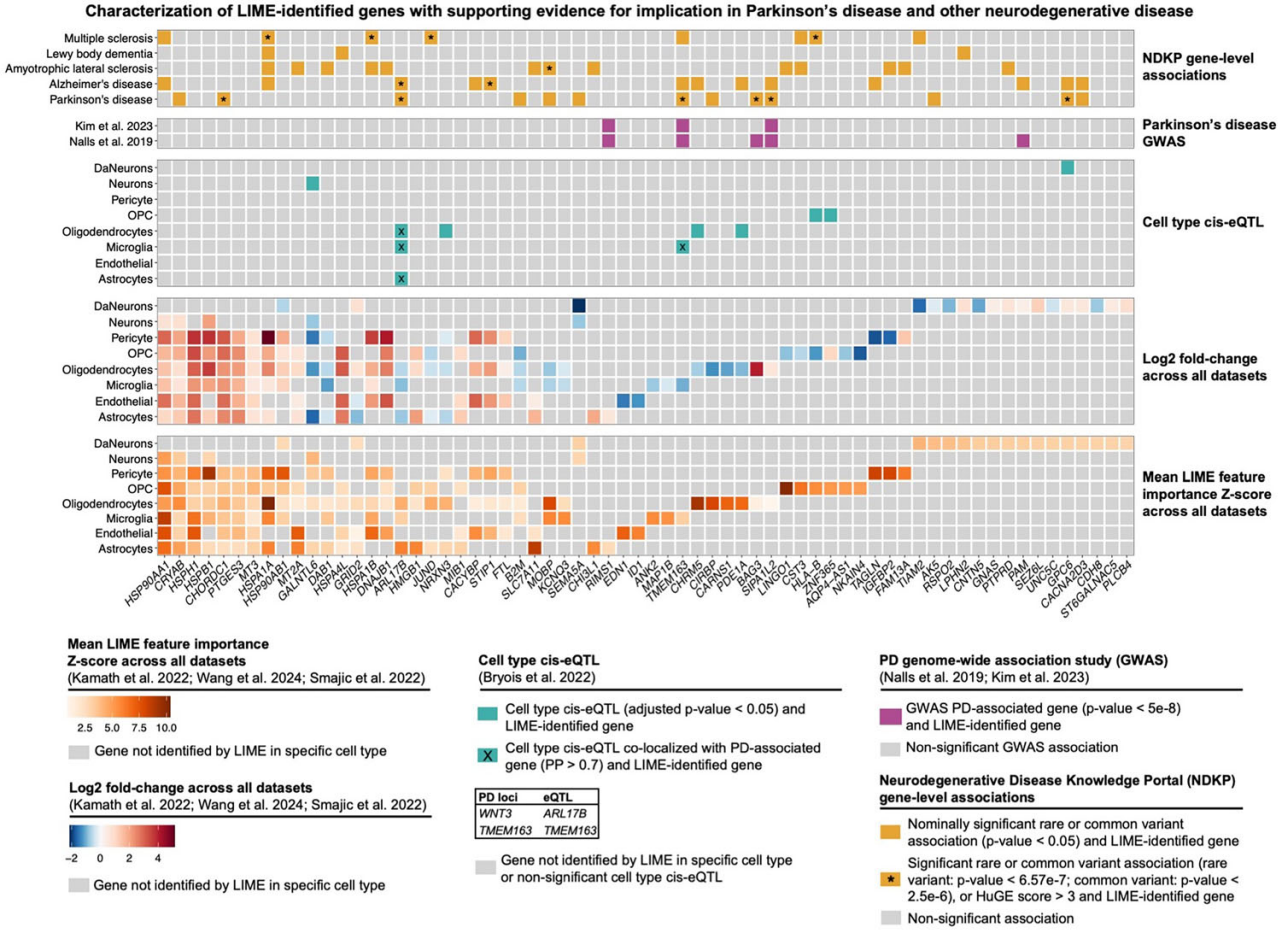
Characterization of filtered LIME-identified genes. Heatmap characterizing the 66 unique genes included in the final LIME-identified gene set. The panels are arranged from bottom to top as follows: 1) Mean LIME feature importance Z-Scores: This panel shows the mean LIME feature importance Z-scores across all snRNAseq datasets. Grey tiles indicate genes that were not identified by LIME in the corresponding cell type. 2) Log2 fold-change: this panel shows the log2 fold-change between Parkinson disease (PD) and control cells across all snRNAseq datasets. Grey tiles indicate genes that were not identified by LIME in the corresponding cell type. 3) Cell type cis-expression quantitative trait loci (eQTL): this panel highlights LIME-identified genes that were identified as cell type cis-eQTLs by Bryois et al. Tiles marked with an “X” represent cell type cis-eQTLs that co-localized with PD-associated genes (posterior probability (PP) > 0.7). Grey tiles indicate genes that were not identified by LIME or were not identified as cis-eQTLs in the corresponding cell type. 4) PD genome-wide association analysis (GWAS): this panel highlights LIME-identified genes that showed an association with PD-risk by GWAS performed by Nalls et al. or Kim et al. Only genes that met genome wide significance (*P* < 5.00e-8) are shown. Grey tiles indicate genes that were not found to be significantly associated with PD in Nalls et al. (bottom) or Kim et al. (top). 5) Neurodegenerative Disease Knowledge Portal (NDKP) gene-level associations: this panel highlights LIME-identified genes that showed gene-level associations with various diseases in the NDKP cohorts. All nominally significant common and rare variant associations are shown. Tiles marked with an asterisk denote significant associations with the corresponding disease or a Human Genetic Evidence (HuGE) Score ≥ 3. Grey tiles indicate non-significant associations. DaNeurons dopaminergic neurons, OPC oligodendrocyte precursor cells.

To further evaluate the biological relevance of LIME-identified genes, we examined public genetic databases for associations with PD and other neurodegenerative diseases. First, using brain cell type-specific cis-expression quantitative trait loci (eQTL) from Bryois et al. ^32^, we found nine genes identified by LIME with significant cis-eQTLs in the corresponding cell type: *GALNTL6* (neurons), *ARL17B* (astrocytes, microglia, and oligodendrocytes), *NRXN3* (oligodendrocytes), *TMEM163* (microglia), *CHRM5* (oligodendrocytes), *PDE1A* (oligodendrocytes), *HLA-B* (OPC), *ZNF365* (OPC), and *GPC6* (DaNeurons) (Fig. 5; Supplementary Table 6) Among these, *TMEM163* co-localized with PD-associated GWAS variants within the *TMEM163* locus, while *ARL17B* co-localized with *WNT3*—another gene linked to PD risk—in the same respective cell types in which they were identified by LIME (Supplementary Table 7)^32^. *TMEM163* and *ARL17B* are particularly noteworthy, as they highlight plausible mechanisms by which PD risk genes may contribute to pathogenesis through dysregulated expression. Their identification by LIME in specific cell types further suggests potential cell type-specific mechanisms driving neurodegeneration in PD.

We next examined PD GWAS data from Nalls et al. ^30^. and Kim et al. ^31^. to identify LIME-identified genes previously associated with PD risk. Five previously identified GWAS genes were uniquely identified in a single cell type by LIME (Supplementary Table 8). *RIMS1* (astrocytes; Nalls et al.: *P* = 2.00e-10, beta = 0.0657; Kim et al.: *P* = 7.00e-12, beta = 0.0208) and *TMEM163* (microglia; Nalls et al.: *P* = 5.00e-14, beta = 0.0807; Kim et al.: *P* = 5.00e-12, beta = 0.0400) showed decreased expression in PD in the snRNAseq midbrain datasets, while *BAG3* (oligodendrocytes; Nalls et al.: *P* = 2.00e-11, beta = 0.0763), *SIPA1L2* (oligodendrocytes; Nalls et al.: *P* = 7.00e-17, beta = 0.1114; Kim et al.: *P* = 1.00e-17, beta = 0.0269) and *PAM* (DaNeurons; Nalls et al.: *P* = 2.00e-9, beta = 0.0621) showed increased expression (Fig. 5). Similarly, we utilized the Neurodegenerative Disease Knowledge Portal (NDKP) database to explore gene-level associations of LIME-identified genes and various neurodegenerative diseases, including PD, Alzheimer disease (AD), amyotrophic lateral sclerosis (ALS), Lewy body dementia (LBD), and multiple sclerosis (MS)^33^. More than half the prioritized LIME gene list showed at least a nominal association with a neurodegenerative disease and six genes had significant common or rare variants associated with PD (Fig. 5; Supplementary Table 9-10). Notably, *ARL17B* showed a statistically significant, common variant association with both PD (Multi-marker Analysis of GenoMic Annotation [MAGMA] *P* = 1.03e-8, Z-statistic = 5.61) and AD (MAGMA *P* = 1.53e-6, Z-statistic = 4.67). Gene burden analyses revealed that individuals with PD were significantly enriched for rare variants in *CHORDC1* (*P* = 6.78e-8, beta = 8.73) and *GPC6* (*P* = 7.67e-8, beta = 16.46).

Based on these results, we compiled a list of 15 LIME-identified genes that exhibit the strongest support for involvement in PD (Table 2). These results highlight the strength of our LIME-based approach in nominating relevant genes for targeted genomic analyses, as evidenced by its ability to identify known PD-associated genes and nominate novel candidate genes.

**Table 2:** LIME-identified genes with supporting evidence for implication in Parkinson disease

Gene	Cell type identified by LIME	Supporting evidence
*AK5*	DaNeurons	•Nominally significant gene-level rare variant association with PD (*p*-value = 4.25e-2, beta = 6.87)
*ARL17B*	Astrocytes, microglia, oligodendrocytes	•Significant gene-level common variant association with PD (MAGMA *p*-value = 1.03e-8, z-statistic = 5.61) •Cell type cis-eQTL co-locolization with *WNT3* (astrocytes: PP = 0.98, beta = 1.22; microglia: PP = 0.98, beta = 1.29; oligodendrocytes: PP = 0.98, beta = 1.41)
*BAG3*	Oligodendrocytes	•GWAS PD-associated gene (Nalls et al. 2019: *p*-value = 2.00e-11, beta = 0.0763)
*B2M*	Microglia, oligodendrocytes, OPC	•Nominally significant gene-level common variant association with PD (MAGMA *p*-value = 2.55e-3, z-statistic = 2.8)
*CACNA2D3*	DaNeurons	•Nominally significant gene-level rare variant association with PD (*p*-value = 1.70e-3, beta = 3.77)
*CHORDC1*	Astrocytes, endothelial, microglia, oligo., OPC, pericytes	•Significant gene-level rare variant association with PD (p-value = 6.78e-8, beta = 8.73)
*CIRBP*	Oligodendrocytes	•Nominally significant gene-level common variant association with PD (MAGMA *p*-value = 3.03e-2, z-statistic = 1.88)
*CRYAB*	Astrocytes, endothelial, microglia, oligo., OPC, pericytes, neurons	•Nominally significant gene-level common variant association with PD (MAGMA *p*-value = 2.96e-2, z-statistic = 1.89)
*GPC6*	DaNeurons	•Significant gene-level rare variant association with PD (*p*-value = 7.67e-8, beta = 16.46)
*MOBP*	Microglia, oligodendrocytes	•Nominally significant gene-level common variant association with PD (MAGMA *p*-value = 9.27e-3, z-statistic = 2.35)
*PAM*	DaNeurons	•GWAS PD-associated gene (Nalls et al. 2019: *p*-value = 2.00e-09, beta = 0.0621)
*SEMA5A*	Neurons, DaNeurons	•Nominally significant gene-level rare variant association with PD (*p*-value = 2.23e-3, beta = 4.62)
*SIPA1L2*	Oligodendrocytes	•GWAS PD-associated gene (Nalls et al. 2019: *p*-value = 7.00e-17, beta = 0.1114; Kim et al. 2023: *p*-value = 1.00e-17, beta = 0.0269)
*RIMS1*	Astrocytes	•GWAS PD-associated gene (Nalls et al. 2019: *p*-value = 2.00e-10, beta = 0.0657; Kim et al. 2023: *p*-value = 7.00e-12, beta = 0.0208)
*TMEM163*	Microglia	•GWAS PD-associated gene (Nalls et al. 2019: *p*-value = 5.00e-14, beta = 0.0807; Kim et al. 2023: p-value = 5.00e-12, beta = 0.0400) •Nominally significant gene-level common variant association with PD (MAGMA p-value = 1.81e-3, z-statistic = 2.91) •Cell type cis-eQTL co-locolization with *TMEM163* (microglia: PP = 0.99, beta = -0.47)

*DaNeurons* dopaminergic neurons, *eQTL* expression quantitative trait loci, *GWAS* genome-wide association study, *MAGMA* Multi Marker Analysis of Genomic Annotation, *PP* posterior probability, *OPC* oligodendrocyte precursor cell, *PD* Parkinson disease.

### Expression and genetic evidence support *GPC6* as a novel PD-associated gene

**Fig. 6:**
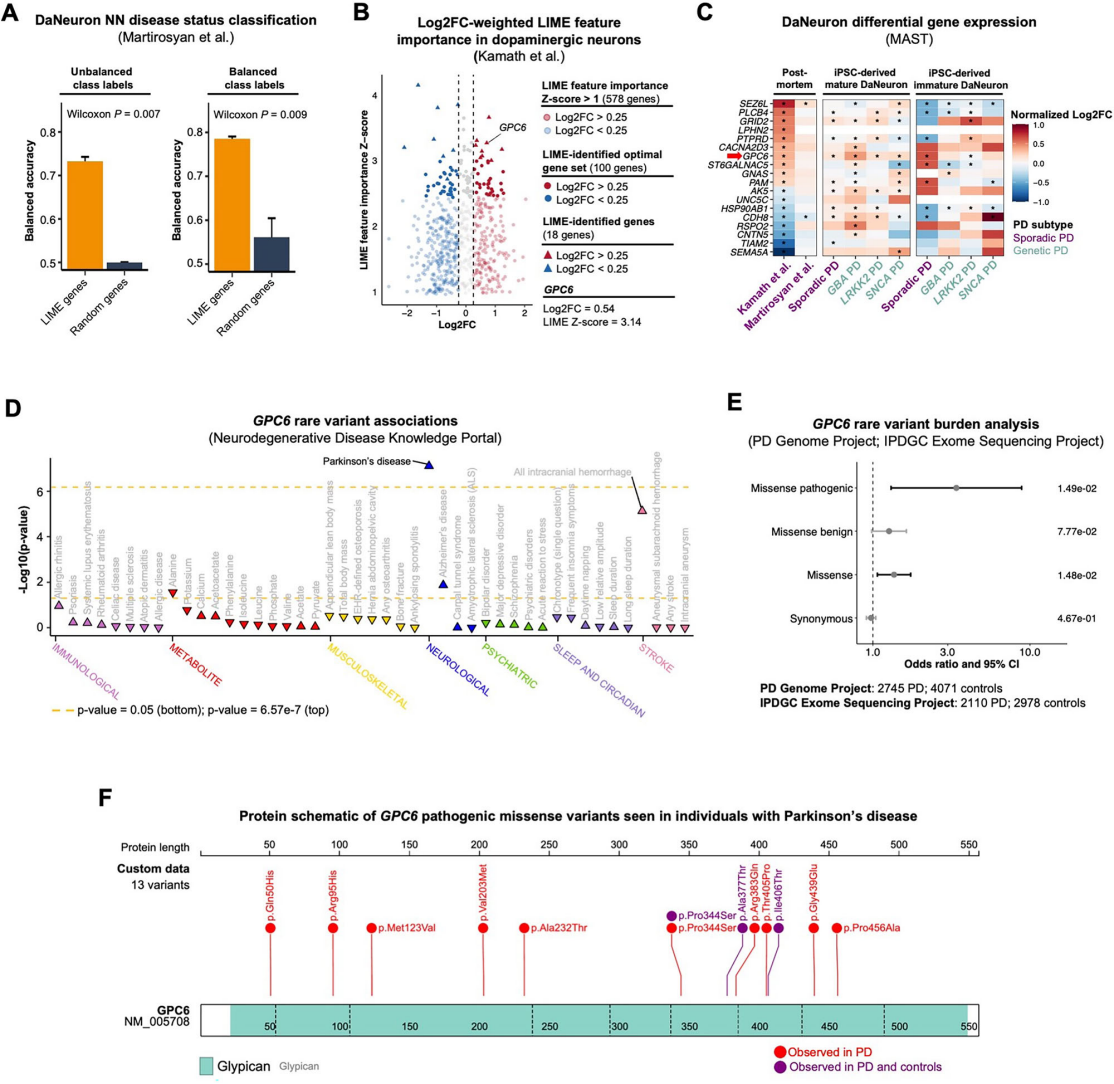
Aberrant expression and rare variant enrichment implicate *GPC6* in Parkinson disease. **A** Bar plot showing the neural network (NN) disease classification balanced accuracy for dopaminergic neurons (DaNeurons) from an independent dataset prepared by Martirosyan et al. LIME-identified genes obtained from Kamath et al. or an equal number of randomly selected genes were input to the classifiers without (left) or with (right) balanced cell counts. Error bars represent the standard deviation of the balanced accuracy across three independent test-train permutations. For each permutation, we used distinct sets of randomly selected genes. Wilcoxon rank-sum tests were used to compare model performance when using the LIME-identified genes versus randomly selected genes. **B** Volcano plot showing the LIME feature importance Z-score and the gene expression log2 fold-change (Log2FC) between Parkinson disease (PD) and control DaNeurons from the post-mortem substantia nigra (SN). Only highly variable genes (HVG) input to the NN classifier with a LIME feature importance Z-score > 1.00 are shown. **C** Heatmap showing the Log2FC of the LIME-identified genes in DaNeurons from the post-mortem SN and induced pluripotent stem cell- (iPSC) derived DaNeurons. Asterisks denote significantly differentially expressed genes (adjusted *P* < 0.05) between individuals with PD and controls computed by MAST. **D** Dot plot showing *GPC6* rare variant associations with various diseases across the Neurodegenerative Disease Knowledge Portal (NDKP) cohorts. **E** Cochran-Mantel-Haenszel (CMH) tests investigating whether individuals with PD from the PD Genome Project and International Parkinson Disease Genomics Consortium (IPDGC) Exome Sequencing Project cohorts were enriched for rare variants in *GPC6*. P-values corresponding to each variant category are shown on the right of the plot. The sample sizes for each cohort are shown below the plot. **F** Protein schematic showing the *GPC6* pathogenic missense variants observed uniquely in PD or in both PD and controls across both cohorts.

Importantly, the Wang et al. and Smajic et al. datasets contained insufficient DaNeurons for NN-LIME analysis, preventing evaluation of the transferability of the LIME-identified gene set (*n* = 118). To address this, we used a fourth dataset from Martirosyan et al., which profiled 2299 DaNeurons from the SNpc of PD and control individuals (Supplementary Fig. 27). Although the dataset was imbalanced (*n* PD = 542; *n* control = 1757) and showed subject-level discrepancies (Supplementary Fig. 27), balancing the cell counts yielded a mean NN balanced accuracy of 0.786 using the LIME genes—significantly higher than models using random genes (mean = 0.546; Wilcoxon *P* = 9.00e-3) (Fig. 6A). Thus, despite the challenges of analyzing DaNeurons without prior neuronal enrichment, the genes identified by LIME effectively distinguished PD cells from a separate dataset, underscoring the generalizability of the identified gene set.

Among the 4349 HVGs originally input to the NN classifier, *GPC6* ranked in the 99.5th percentile for distinguishing PD from healthy DaNeurons, the cell type lost in PD pathology and thus of particular interest (Fig. 6B). Given the high LIME feature importance, enrichment of rare variants in PD cases from the NDKP cohort, and its identification as a cis-eQTL in neurons of the adult human brain, we prioritized *GPC6* for further investigation.

In post-mortem midbrain tissue, *GPC6* showed elevated expression in PD DaNeurons from both datasets (Fig. 6C). However, a major challenge in transcriptomics is distinguishing between gene expression changes driven by genetic factors, environmental exposures, or a response to the disease process itself. Furthermore, while post-mortem tissues provide valuable insights into the late stages of disease, they do not capture disease onset and progression. Using scRNAseq on patient-derived induced pluripotent stem cells (iPSC) mitigates these limitation since environmental factors and pathophysiology-induced transcriptional changes are unlikely to persist after iPSC differentiation, revealing molecular signatures driven by the underlying genetic architecture of patient-derived cells^34^. We therefore analyzed scRNAseq data from iPSC-derived mature and immature DaNeurons, and compared apparently sporadic PD (*n* = 29) or genetic PD (*GBA* [*n* = 16], *LRRK2* [*n* = 22], and *SNCA* [*n* = 3]) with controls (*n* = 11)^35^. Notably, *GPC6* expression was significantly increased in sporadic PD and across all genetic subtypes compared to controls in mature DaNeurons (Fig. 6C). DGE analysis in the immature DaNeurons revealed a significant increase in *GPC6* expression in sporadic PD patients compared to controls, but not in individuals with genetic PD (Fig. 6C). The increased expression of *GPC6* across datasets, experimental systems, and PD subtypes supports a role in PD and underscores its potential as a disease marker.

To further interrogate the enrichment of *GPC6* rare variants in individuals with PD that we observed in the NDKP cohorts (Fig. 6D), we leveraged two additional case-control cohorts: the PD Genome Project (*n* PD = 2745; *n* control = 4071) and the International Parkinson Disease Genomics Consortium (IPDGC) Exome Sequencing Project (*n* PD = 2,110; *n* control = 2,978) for rare variant gene burden analyses^36^. While individual Fisher’s exact tests showed enrichment of damaging missense variants in PD cases, these results did not meet statistical significance (PD Genome Project: *P* = 7.80e-2, odds ratio [OR] = 2.97; IPDGC: *P* = 7.32e-2, OR = 4.24) (Supplementary Fig. 28). However, a combined Cochran-Mantel-Haenszel (CMH) test across both cohorts revealed a significant enrichment (*P* = 1.49e-2, OR = 3.40) (Fig. 6E). Figure 6F shows the pathogenic missense variants observed in individuals with PD across both cohorts. The identification of *GPC6* in DaNeurons by LIME, its aberrant expression in both post-mortem tissue and in vitro models, and the enrichment of damaging variants in individuals with PD across three cohorts collectively support its involvement in PD.

## Discussion

Identifying genetic contributors to disease is essential for advancing therapeutic development; yet, much of the heritability in complex diseases like PD remains unexplained^9^. With its single-cell resolution, scRNAseq is uniquely positioned to nominate aberrantly expressed, cell type-specific genes for targeted genomic analyses to uncover novel genetic associations. Realizing this potential, however, requires identifying consistent disease-associated markers—an effort that is insufficiently addressed by current DGE methods. To address this, we introduced a framework that trains ML models to classify single-nuclei transcriptomes by disease status and uses an interpretable model explainer to identify genes driving these classifications.

Our framework offers key advantages over traditional statistical methods for identifying gene expression changes in diseased cells. First, unlike standard DGE, which relies on arbitrary P-value and fold-change thresholds and often yields overwhelming numbers of significant genes, our ML workflow directly prioritizes a minimal, high-impact gene set. Through permutation testing with varying feature counts, it empirically optimizes the balance between model performance and input size, streamlining gene selection and enhancing biological interpretability in an unsupervised manner. We note that our analysis applied Z-score and fold-change cut-offs to further refine the LIME optimal gene sets, in alignment with the study’s primary goal of nominating novel candidate genes in PD. Nonetheless, this step is independent of the machine learning framework itself and serves merely as one example of a downstream analysis of the LIME-identified gene sets, which will necessarily vary across studies. Second, our framework facilitates pan-dataset analyses and enables direct evaluation of marker transferability across datasets, mitigating the limitations posed by small sample sizes and facilitating cohort-scale studies needed to address the heterogeneity of complex diseases like PD.

A key finding of this study was the identification of cell type-specific LIME optimal gene sets that accurately classified PD cells across datasets. However, we note an important distinction between transferable molecular markers and generalizable models. While generalizable models—capable of classifying cells across datasets with a single NN—are essential for clinical applications like diagnosis, they offer limited value for post-mortem samples, which are typically accompanied by detailed clinical metadata. As such, this application was beyond the scope of the present study. Instead, we focused on transferable molecular markers: genes whose expression enables high classification accuracy when used as input to dataset-specific models. Indeed, the transferability of the LIME-identified genes from this study suggests that they captured replicable readouts across datasets, which is critical for establishing their relevance to PD.

Overrepresentation analysis of the LIME optimal gene sets revealed enrichment of biological processes well-established in PD, confirming their biological relevance beyond in silico classification. Genes related to the heat shock and unfolded protein response were prominently enriched across non-neuronal cell types, reflecting the accumulation of α-synuclein aggregates in PD^2^. While pathway-level analyses generate hypotheses about the pathophysiology of neurodegeneration, gene-level approaches are critical for identifying drivers of disease. To this end, we used a reductionist strategy to highlight the most informative LIME-identified genes. Intriguingly, several genes known to cause distinct monogenic neurological disorders—*DAB1* (spinocerebellar ataxia 37)^37^, *GRID2* (spinocerebellar ataxia 18)^38^, and *FTL* (neuroferritinopathy)^39^—were influential in classifying PD cells, reinforcing emerging evidence for shared mechanisms among neurological disorders^40^. LIME also recovered known PD GWAS genes (*RIMS1*, *TMEM163*, *BAG3*, *SIPA1L2*, and *PAM*), validating its utility for nominating candidate genes. Interestingly, these genes were uniquely identified by LIME in distinct cell types, suggesting cell type-specific mechanisms of the PD-associated genes. If future studies can support the involvement of the LIME-identified PD-associated genes in specific cell types, as suggested by this analysis, it would enable us to substantially refine our mechanistic hypotheses regarding the etiology of PD.

Beyond these previously established genetic drivers, Table 2 presents an additional ten candidate genes identified by LIME, which exhibited varying levels of genetic support for their involvement in PD and thus merit further investigation. *ARL17B*—implicated in protein trafficking and vesicle-mediated transport—is of particular interest. Identified by LIME in astrocytes, microglia, and oligodendrocytes, *ALR17B* showed statistically significant common variant associations with both PD and AD from the NDKP cohorts^33^. Its expression also co-localized with PD-associated variants in *WNT3* within these same cell types^32^. Moreover, transcriptome-wide association analysis linked decreased *ARL17B* expression to increased PD risk^41^, further supporting its potential role in disease pathology.

Among the most compelling novel genes identified by LIME was *GPC6*, a glycosylphosphatidylinositol-anchored heparan sulfate proteoglycan uniquely detected in DaNeurons. *GPC6* was upregulated in both DaNeurons from post-mortem midbrain tissue and iPSC-derived models of mature DaNeurons from individuals with apparently sporadic PD and genetic PD compared to controls, suggesting a shared pathogenic role in late stage-disease across PD subtypes. Notably, *GPC6* was significantly upregulated in immature iPSC-derived DaNeurons only in sporadic PD, suggesting an early and potentially contributory role in polygenic disease. This contrasts with genetic cases, where elevated *GPC6* expression was observed only in mature DaNeurons, implying it may be a downstream consequence of pathogenic variants in *GBA*, *SNCA*, and *LRRK2*. The consistent upregulation of *GPC6* across the DaNeuron maturation axis in sporadic PD, together with the enrichment of rare variants in PD cases across three independent cohorts, suggests a potential role for *GPC6* in the complex interplay of moderate-effect variants contributing to PD risk. Heparan sulfate proteoglycans are known to mediate intracellular accumulation and propagation of α-synuclein fibrils, implicating them in disease progression across synucleinopathies^42^. One hypothesis is that DaNeurons, in response to *GPC6* loss of function or dysfunction, upregulate related proteoglycans—including *GPC6* itself through feedback mechanisms—to enhance α-synuclein fibril uptake. Supporting this, *GPC6* has been identified as a cis-eQTL that increases expression in neurons^32^. Alternatively, studies in animal models suggest that glycosylphosphatidylinositol-anchored proteoglycans are critical for synaptic function^43^, and that phospho-α-synuclein-positive neurons show elevated *GPC6* expression in PD models^44^. Thus, aberrant *GPC6* may contribute to synaptic dysfunction and DaNeuron degeneration. Of course, these mechanisms are not mutually exclusive, as synaptic dysfunction driven by α-synuclein aggregation is a hallmark of PD^24^. Fig. 6F presents the pathogenic missense variants identified in individuals with PD from the PD Genome Project and IPDGC Exome Sequencing Project cohorts, providing a valuable foundation for future functional studies aimed at further exploring the role of *GPC6* in PD.

A key limitation of this study is the reliance on post-mortem tissue, which captures a snapshot of late-stage disease and cannot provide insight into disease onset or progression. Although we examined the expression of LIME-identified genes across the maturation axis of iPSC-derived DaNeurons in culture to infer their relevance to early-stage PD, more sophisticated perturbation models will be essential to validate and further investigate their roles in disease progression. An additional limitation is that our analysis prioritized top candidate genes based on scRNAseq data and statistical genetics approaches, underscoring the need for functional validation through orthogonal lines of evidence to support their involvement in PD.

We anticipate that this framework for identifying molecular markers of disease will be broadly applicable across diseases. While we used a reductionist approach to prioritize biologically relevant genes, LIME outputs remain flexible and customizable. In this proof-of-concept study, we focused on HVGs, though this inherently limits discovery to a subset of genes. Interpretable dimensionality reduction techniques can mitigate this limitation but require a careful balance between discovery power and classifier noise. Overall, we conclude that our ML framework offers a promising opportunity to resolve the complex genetic architecture of polygenic diseases like PD.

## Methods

### Data acquisition, pre-processing, and cell type annotation

We analyzed four snRNAseq datasets profiling the post-mortem midbrain of individuals with PD and controls from Kamath et al. ^10^, Wang et al. ^11^, Smajic et al. ^12^, and Martirosyan et al. ^45^. Publicly available feature-barcode expression matrices and metadata were sourced from their respective data repositories. LBD samples (Kamath et al.) and PD samples with frontotemporal dementia (Wang et al.) were excluded. To corroborate the snRNAseq data, we utilized a scRNAseq dataset generated by Bressan et al. ^35^ that profiled mature and immature iPSC-derived DaNeurons from individuals with apparently sporadic PD—those without known pathogenic variants in genes associated with genetic PD—as well as from individuals with genetic PD carrying *GBA* N370S, *LRRK2* G2019S, *LRRK2* R1441G, or *SNCA* A53T mutations, and from controls. A processed Seurat object was filtered to include only DaNeurons from cultures differentiated for 65 days.

Pre-processing and quality control of the post-mortem snRNAseq data were performed using Seurat (v4.3.0.1)^16,46^. Cells were filtered based on the number of unique features and unique molecular identifiers per cell, adhering to the thresholds utilized in the original manuscripts (Table 3)^10–12,45^. Cells with > 10% mitochondrial reads or ribosomal proteins were excluded. Putative doublets were detected with DoubletFinder (v2.0.4) and removed^47^. Cells were clustered using Louvain network detection. Cluster marker genes were identified using the Wilcoxon rank-sum test (log2FC > 0.25, *P* < 0.05), then analyzed for enrichment of known cell type markers using EnrichR^48^ and compared against literature-curated cell type markers for cluster annotation. Cell type similarity across datasets was evaluated with MetaNeighbor (v1.18.0)^49^.

**Table 3:** Description of data sources and filtering conditions

Dataset	Description	Source	Filtering thresholds
Kamath et al. ^10^	snRNAseq human midbrain	https://singlecell.broadinstitute.org/single_cell/study/SCP1768	Minimum UMI per cell: 650
Wang et al. ^11^	snRNAseq human midbrain	GEO; GSE184950	Minimum features per cell: 200; maximum features per cell: 2500
Smajic et al. ^12^	snRNAseq human midbrain	GEO; GSE157783	Minimum UMI per cell: 1500; minimum features per cell: 1000
Martirosyan et al. ^45^	snRNAseq human midbrain	GEO; GSE243639	Minimum UMI per cell: 500
FOUNDIN-PD; Bressan et al. ^35^	iPSC-derived DaNeurons	www.ppmi-info.org/access-dataspecimens/download-data; SCR 006431	Minimum features per cell: 1000; maximum features per cell: 9000

Cells were filtered according to the thresholds defined in the corresponding manuscripts.

*GEO* Gene Expression Omnibus, *snRNAseq* single nuclei RNA sequencing, *UMI* unique molecular identifier.

### Machine learning models for disease classification

Individual AnnData objects were generated for each cell type to enable analyses using the Scanpy (v1.9.2) library in Python (v3.8.10). Each dataset was split 80/20 for training and testing. Hyperparameters were optimized using five-fold cross-validation on the training data, with models evaluated on the held-out test set. We used the scikit-learn Python library to implement four different ML classifiers. (i) NN was implemented with *MLPClassifier*, using one hidden layer (100 nodes), ReLu activation, Adam optimizer, and a 500-iteration cap. (ii) LR was implemented with *LogisticRegression*, using L1 regularization (strength = 2.0), liblinear solver, and a 100-iteration cap. (iii) RF was implemented with *RandomForestClassifier* using 100 trees, Gini impurity, minimum sample split = 2, minimum leaf = 1, and bootstrapping enabled. (iv) SVM was implemented using *SVC* with a linear kernel, regularization strength = 1, and no iteration limit.

### Feature selection for input to machine learning classifiers

SnRNAseq counts were normalized and log-transformed using Scanpy. To preserve test set independence, feature selection was conducted on the training split and applied independently to the testing split. We evaluated four feature selection methods. (i) HVG were identified using Scanpy’s *highly_variable_genes* function and the *Seurat* flavor, which selects genes based on normalized dispersion without requiring the user to predefine the number of genes. The single-cell expression levels of the HVGs were input to the ML models. (ii) PCA was performed using scikit-learn (v1.2.1)^50^. The optimal number of components was determined using Seurat’s *JackStraw* method, retaining all components up to the first with *P* ≥ 0.05. (iii) NMF was performed using scikit-learn (v1.2.1)^50^. To select the optimal number of components, we varied the number of components (1–100), calculated reconstruction error, and identified the elbow point using the Unit Invariant Knee method via the *inflection* (v1.3.6) R package^51^. (iv) ETM was performed using scETM with default parameters and 100 epochs^52^. To select the optimal number of topics, we trained models with 10–100 topics and selected the topic count that yielded the highest average classification accuracy. For methods ii-iv, the cell-by-component matrices were input to the ML models.

### Machine learning classifier performance evaluation

We evaluated the performance of the ML classifiers on 20% of the unseen data reserved for testing using balanced accuracy:

1 $${Balanced\; Accuracy}=\frac{1}{2}\left(\frac{TP}{TP+FN}+\frac{TN}{TN+FP}\right)$$

where ${TP}$ denotes the number of cells correctly classified as PD; ${TN}$ denotes the number of cells correctly classified as healthy control; ${FP}$ denotes the number of cells incorrectly classified as PD; and ${FN}$ denotes the number of cells incorrectly classified as healthy control. Additionally, we performed leave-one-subject-out analyses to assess the generalizability of the ML classifiers: cell type-specific models were trained on all subjects except one, which was held out for evaluation. This process was iterated to ensure that every subject was used as the evaluation set.

### Application of LIME to machine learning classifiers

We developed a custom approach that leverages LIME outputs from the *lime* Python library to interpret the ML classifiers and identify the most important genes driving the classification of a cell as PD^19^. For each cell in the test set, LIME returned a ranked list of all features input to the ML model ordered according to their influence on the classification decision. To approximate global explanations from LIME, we calculated the average feature importance of each gene across correctly classified cells belonging to a PD subject:

2 $${F}_{i,j}=\frac{1}{{N}_{i}}\mathop{\sum }\limits_{j=1}^{{N}_{i}}|{{LIME}}_{i,j,k}|$$

where ${F}_{i,j}$ represents the average absolute feature importance for gene $j$ in subject $i$, ${N}_{i}$ is the number of test cells for that subject, and ${{LIME}}_{i,j,k}$ is the LIME feature importance for gene $j$ in the $k$-th cell of subject $i$. Next, we averaged these values across all PD subjects:

3 $${F}_{j}=\frac{1}{M}\mathop{\sum }\limits_{i=1}^{M}{F}_{i,j}$$

where $M$ is the number of PD subjects. To reduce the likelihood of selecting sparsely expressed genes and to favor candidates with greater potential for generalization across datasets, we weighted the LIME feature importance by normalized percent expression. We first computed the percent expression of each feature as:

4 $${P}_{j}=\frac{{C}_{j}}{C}\times 100$$

where ${P}_{j}$ is the percent expression of gene $j$, ${C}_{j}$ is the number of cells expressing gene $j$, and $C$ is the total number of cells. We then normalized these percent expression values so that they summed to 1.00:

5 $${N}_{j}=\frac{{P}_{j}}{{\sum }_{l=1}^{L}{P}_{l}}$$

where ${N}_{j}$ is the normalized percent expression of gene $j$ and $L$ is the total number of genes. This normalization step ensures that each gene’s expression is assessed in relation to all other genes and prevents the model from disproportionately favoring highly expressed genes, allowing the LIME importance scores to drive the prioritization after weighting. We weighted the LIME importance scores as:

6 $${W}_{j}={N}_{j}\times {F}_{j}.$$

Finally, we applied a Z-score transformation to standardize the weighted importance score for comparison across datasets:

7 $${Z}_{j}=\frac{{W}_{j}-\mu }{\sigma }$$

where $\mu$ is the mean of the weighted scores and $\sigma$ is the standard deviation of those scores.

### Identification of LIME optimal gene sets

We performed permutation tests using progressively fewer genes input to the ML models to determine the optimal gene sets for disease classification. We assigned percentile ranks to all HVGs based on their mean LIME feature importance Z-score across exploratory datasets (Kamath et al. and Wang et al.). Starting from the percentile rank corresponding to a mean Z-score = 1.00, we trained ML classifiers using all genes meeting this threshold and evaluated its performance. We then incrementally increased the percentile threshold by 0.001, thereby decreasing the number of genes input to the ML model, and re-trained and re-evaluated the classifier. We repeated this process until we reached a percentile rank of 1.00, retaining only the most important genes. We performed the same permutation tests with randomly selected genes as a benchmark. Let ${{ML}}_{{LIME}}$ and ${{ML}}_{{gene}}$ be the balanced accuracy of the ML classifier using LIME-identified genes and randomly selected genes, respectively, and let $\Delta {{ACC}}_{{dataset}}$ be the discrepancy in the balanced accuracy of the ML classifiers when using an equal number of LIME-identified genes or random genes for a give exploratory dataset. Our method to identify the optimal gene set is defined as:

8 $${Optimal\; gene\; set}={\arg \max }_{G}(\Delta {{ACC}}_{{Kamath\; et\; al}.\,}+\Delta {{ACC}}_{{Wang\; et\; al}.\,})$$

where ${{ACC}}_{{dataset}}$ is defined as:

9 $${{ACC}}_{{dataset}}={{ML}}_{{LIME}}-{{ML}}_{{gene}}$$

### Genetic analysis of LIME-identified genes

To characterize the LIME-identified genes, we used publicly available databases with genetic and expression data on PD and other neurodegenerative diseases. Cell type cis-eQTL were obtained from Bryois et al. ^32^. We reported cell type cis-eQTLs with an FDR-adjusted *P* < 0.05 and co-localizations with PD-associated genes with a posterior probability > 0.70, provided the cis-eQTL was identified in the same cell type as the corresponding LIME-identified gene. For LIME-identified genes in neurons and DaNeurons, we reported cis-eQTLs identified in inhibitory and excitatory neurons. PD GWAS summary statistics were obtained from Nalls et al. ^30^ and Kim et al. ^31^. We reported the mapped gene for associations that achieved genome-wide significance (*P* < 5.00e-8). Gene-level association statistics were obtained from NDKP (https://ndkp.hugeamp.org/; 08-05-2024). We investigated common variant associations identified using MAGMA^53^, rare variant gene burden analyses, and Human Genetic Evidence (HuGE) scores^54^ for PD, AD, ALS, LBD, and MS. For common and rare variant associations, we reported all LIME-identified genes with nominally significant associations (*P* < 0.05), highlighting those with a MAGMA *P* < 2.50e-6 and gene burden analyses with *P* < 6.57e-7. HuGE Scores were computed using the HuGE Calculator (https://ndkp.hugeamp.org/hugecalculator.html; 08-05-2024). We reported LIME-identified genes with a HuGE score ≥ 3 (*Moderate* evidence).

### *GPC6* rare variant gene burden analyses in additional case-control cohorts

To assess the enrichment of *GPC6* rare variants in individuals with PD, we performed gene burden analyses using two case-control cohorts: the PD Genome Project and IPDGC Exome Sequencing Project (https://pdgenetics.shinyapps.io/VariantBrowser/; 08-25-2024)^36^. Variants in *GPC6* were annotated using the Variant Effect Predictor (v.112.0)^55^. Coding variants were grouped into three categories: putative loss of function, missense, and synonymous. Due to the presence of only one loss-of-function variant in both cohorts, this variant category was excluded. Missense variants were further classified as damaging if labeled “pathogenic” by AlphaMissense or “ambiguous” with a CADD score >20.000. Only rare variants (allele frequency <0.01 in gnomAD) were retained. We used two-sided Fisher’s exact tests to compare the frequency of rare variants in PD cases versus controls within each variant category, analyzing each cohort independently. A combined analysis was conducted using the CMH test to assess association while controlling for cohort as the stratifying variable^56^.

### Pan-dataset differential gene expression analysis

Cell type-specific DGE was calculated using MAST^57^, the Wilcoxon rank-sum test, and DESeq2^58^, as implemented in the scRNAbox pipeline^16^. MAST and Wilcoxon tests were performed via Seurat’s *FindMarkers* function, which treats each cell as an independent replicate. Additionally, we applied MAST under a mixed-effects framework, specifying subject ID as a random effect using the *latent.vars* parameter. DESeq2 was applied using a pseudo-bulk approach, in which counts from all cells belonging to a given subject were aggregated using Seurat’s *AggregateExpression* function, treating each subject as an independent biological replicate. To minimize false-positive signals, we restricted the analysis to genes expressed in at least 1% of PD or control cells within each cell type. Genes with a Bonferroni-adjusted *P* < 0.05 and an absolute value Log2FC > 1.00 were deemed differentially expressed.

To evaluate the generalizability of signals identified by each DGE method, we performed a cross-dataset intersectional analysis, reasoning that genes consistently detected across independent datasets are more likely to represent robust and actionable signals. Each method was applied separately to the Kamath et al. and Wang et al. datasets, and we calculated the proportion of overlapping DEGs (pan-dataset genes). For NN-LIME, we selected genes with a cell type-specific LIME feature importance Z-score > 1.00 from each dataset and similarly computed the proportion of pan-dataset genes. Finally, we compared the proportions of cell type-specific pan-dataset genes across methods using a Wilcoxon rank-sum test.

### Gene set overrepresentation analysis

Gene set overrepresentation analysis was performed using the g:Profiler web interface (https://biit.cs.ut.ee/gprofiler/gost; 07-22-2024)^59^, configured to use all genes identified in the midbrain datasets as the statistical domain scope. Terms with a g:SCS adjusted *P* < 0.05 were considered significantly overrepresented^60^.

### Statistical analysis

We performed statistical analyses in R^61^ (v4.2.2) and used the ggplot2^62^ (v3.4.2) R package for data visualization. For machine learning development and evaluation, individual cells were randomly partitioned into training and testing sets according to their cell indices. A priori sample size calculations were not conducted, as the analyses utilized publicly available datasets.

## Supplementary information

Supplementary information

Supplementary Tables

## Data Availability

SnRNAseq data prepared by Kamath et al. is available from the Single Cell Portal (https://singlecell.broadinstitute.org/single_cell/study/SCP1768). SnRNAseq data prepared by Wang et al. are available from the GEO with accession code GSE184950. SnRNAseq data prepared by Smajic et al. are available from the GEO with accession code GSE157783. SnRNAseq data prepared by Wang et al. are available from the GEO with accession code GSE184950. SnRNAseq data prepared by Martirosyan et al. are available from the GEO with accession code GSE243639. ScRNAseq data characterizing the iPSC-derived DaNeurons prepared by Bressan et al. are available from the PPMI database (www.ppmi-info.org/access-dataspecimens/download-data), RRID:SCR 006431. For up-to-date information on the study, visit www.ppmi-info.org. The genomics data from the PD Genome Project and IPDGC Exome Sequencing Project are available from the Parkinson Disease Variant Browser (https://pdgenetics.shinyapps.io/VariantBrowser/).

## Code availability

The code used for the analyses presented in the work, as well as the NN-LIME pipeline, is available on GitHub (https://github.com/mfiorini9/scRNAseq-NN-LIME).
